# Supplementary material for: Exploring Semi-Quantitative Metagenomic Studies Using Oxford Nanopore Sequencing: A Computational and Experimental Protocol
Source: Genes (Basel). 2021 Sep 25;12(10):1496. doi: 10.3390/genes12101496 (PMC8536095; doi:10.3390/genes12101496)
Supplement: Supplementary file 1 [file genes-12-01496-s001.zip › supplementary.pdf]

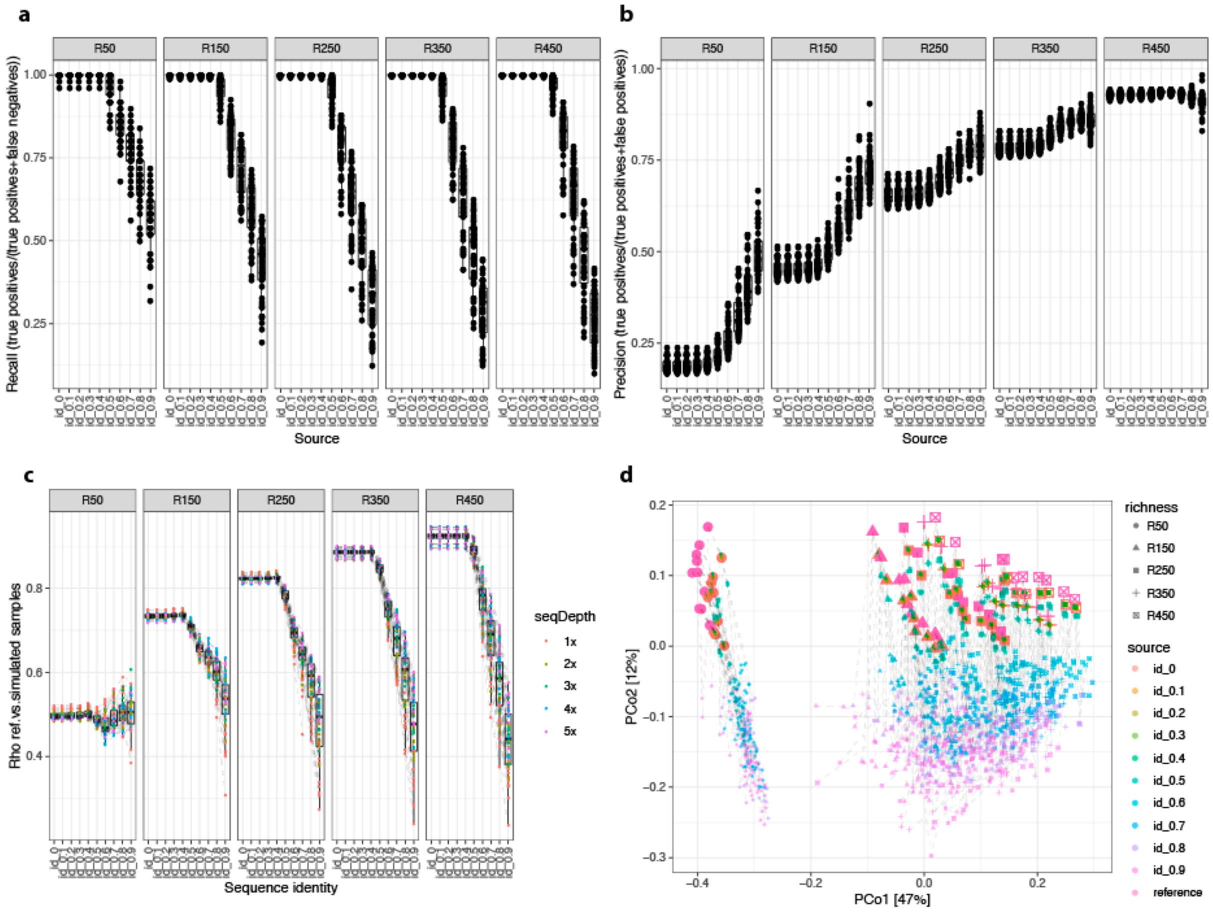

Supplemental Figure S1: Impact of filtering minimap2 primary alignments of ONT reads at different thresholds of sequence identity. Boxplots of recall (a) and precision (b) values of species richness estimates in 250 simulated samples (y-axis) between metagenomic profiles inferred from primary alignments of ONT reads filtered by different thresholds of sequence identity (from 0 to 90%; x-axis) stratified by the number of species in reference metagenomic profiles. (c) Boxplots of Spearman's Rho coefficients in correlation analyzes between taxonomic profiles of reference and simulated samples (y-axis) at different thresholds of sequence identity (from 0 to 90%; x-axis) stratified by the number of species in reference metagenomic profiles. Points are colored according with the sequencing depth of simulated samples. (d) PCoA of reference and simulated samples inferred from minimap2 primary alignments filtered by different thresholds of sequence identity (from 0 to 90%; x-axis). Dashed lines connect points coming from the same sample (reference, simulated ones; 11 points per sample) with different shapes assigned to samples from different reference species richness. Points corresponding to reference samples and simulated samples with no filtering by sequence identity (id_0) are highlighted with larger point sizes.

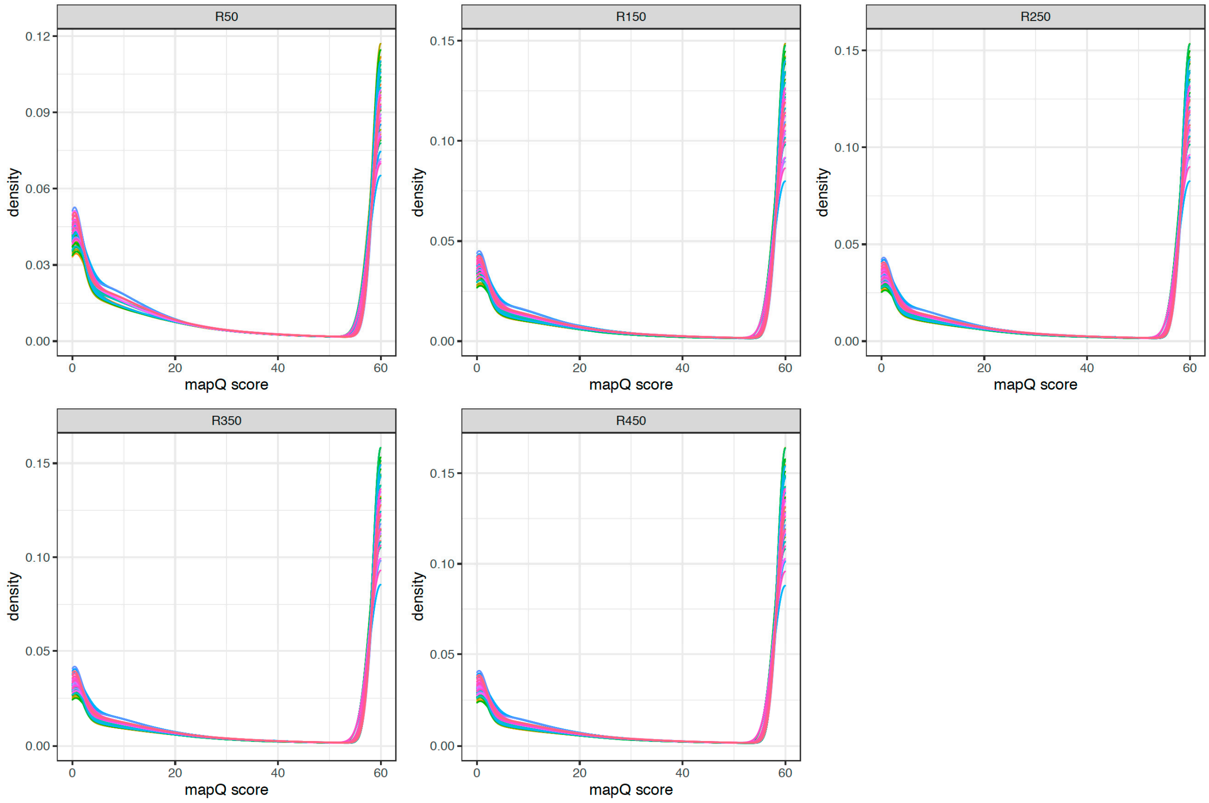

Supplemental Figure S2: Density distributions of mapQ scores in primary alignments of 250 simulated samples stratified by the number of species in reference samples (50 samples per reference species richness).

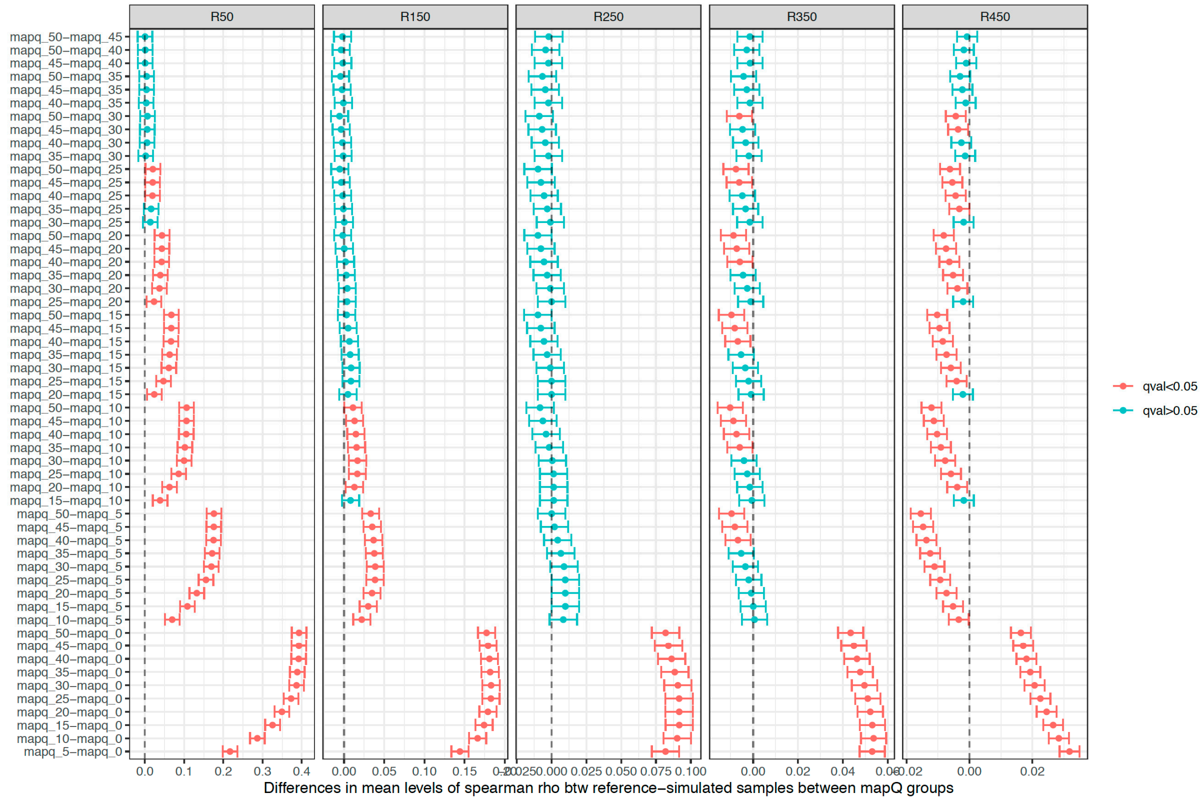

Supplemental Figure S3: Statistical comparison of differences between reference and simulated samples at different thresholds of mapQ scores. At each level of reference species richness (from 50 to 450 species, 50

samples per level), we compare the distributions of the similarities in species abundances between reference and simulated samples (Spearman's Rho coefficients of correlations between reference and simulated species abundance vectors) for all possible pairs of mapQ thresholds evaluated with Trukey's post-hoc pairwise tests. The 95% family-wise confidence level in the difference between pairs of mapQ threshold is represented colored by the significance of the difference according to adjusted P-values in Tukey's tests. If we focus on the mapQ=5, we observe that higher mapQ values leads to higher similarities between reference and simulated species abundance vectors (positive values in the confidence levels of the differences) in R50 and R150 simulated samples, whereas this is not the case for more compex/rich simulated samples (R250-R450), where we observe that the similarities with the reference decrease as we increase the stringency of the mapQ filtering (negative values in the confidence levels of the differences, being significant for R450 samples).

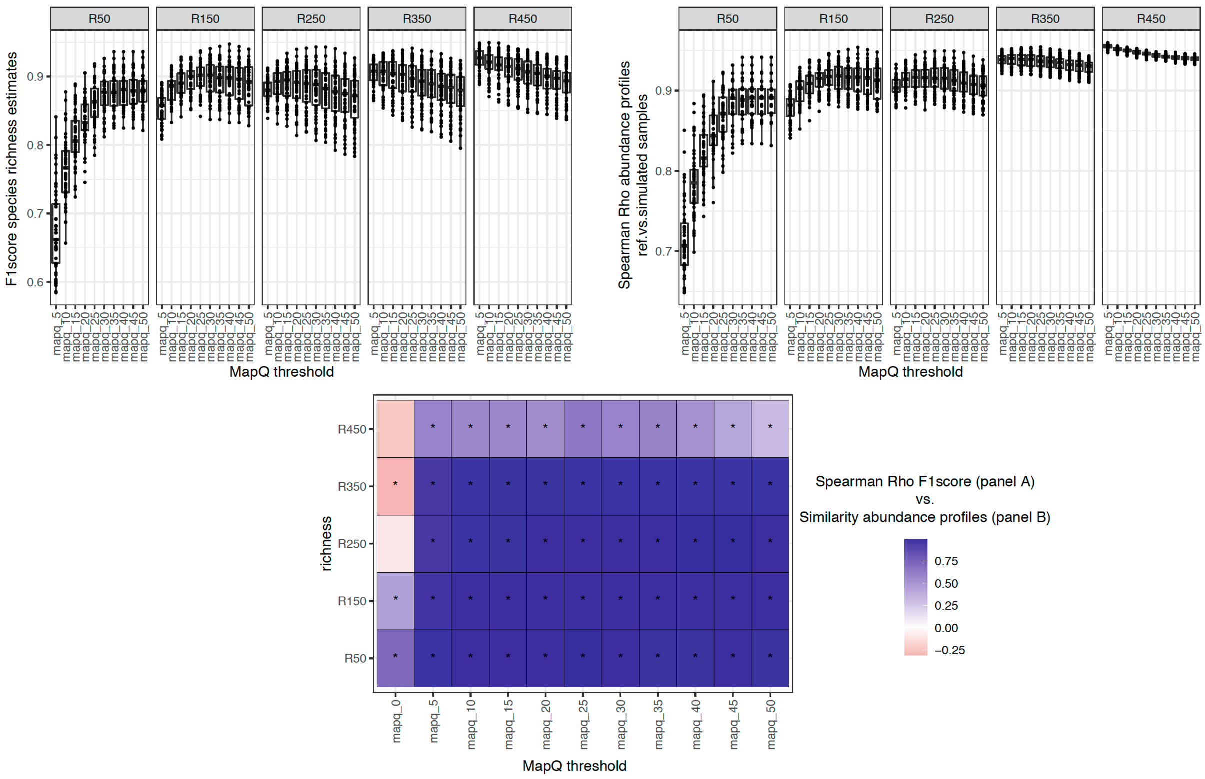

Supplemental Figure S4: Similarity in species richness and species abundances estimations across different mapQ thresholds. (A) Distribution of F1 scores in species richness estimates (harmonic mean of precision and recall; y-axis) across simulated data filtered by different mapQ thresholds (x-axis) and stratified by the complexity of simulated microbial communities. (B) Distribution of similarities between simulated and reference abundance profiles (Spearman Rho's) across simulated data filtered by different mapQ thresholds (x-axis) and stratified by the complexity of simulated microbial communities. (c) Correlogram of Spearman Rho's between F1 scores in panel A and similarities between simulated and reference abundance profiles in panel B. *=P-value<0.05 Spearnan Rank test.

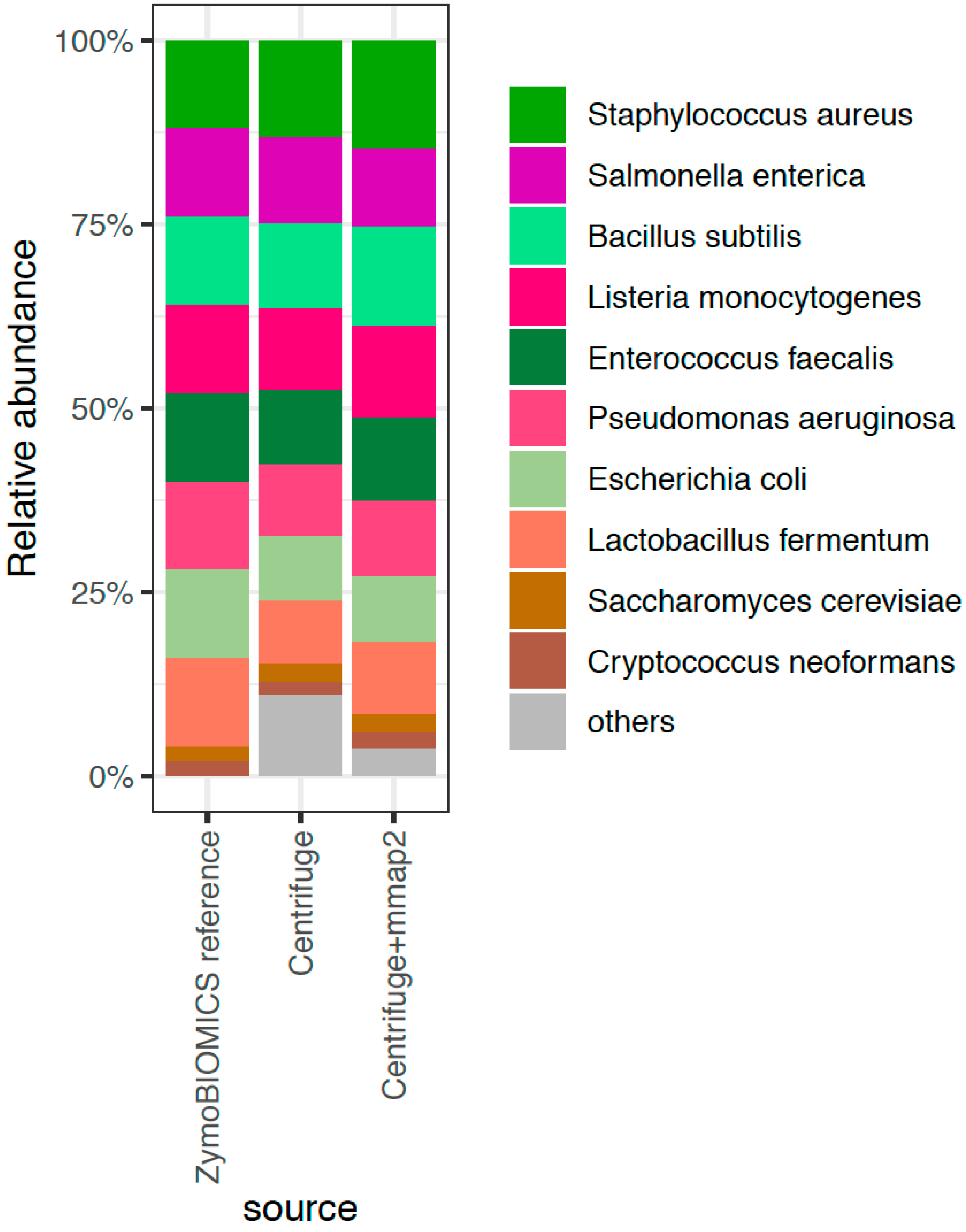

Supplemental Figure S5: Taxonomic profile of ZymoBIOMICS mock community inferred from Nanopore sequencing. The reference composition of ZymoBIOMICS mock community is compared with the taxonomic profile obtained from Nanopore sequencing data with Centrifuge only and with Centrifuge combined with filtering of read bins by minimap2 mapping against the corresponding reference genomes with parameters derived from simulation experiments (primary alignments only, min. mapQ=5)

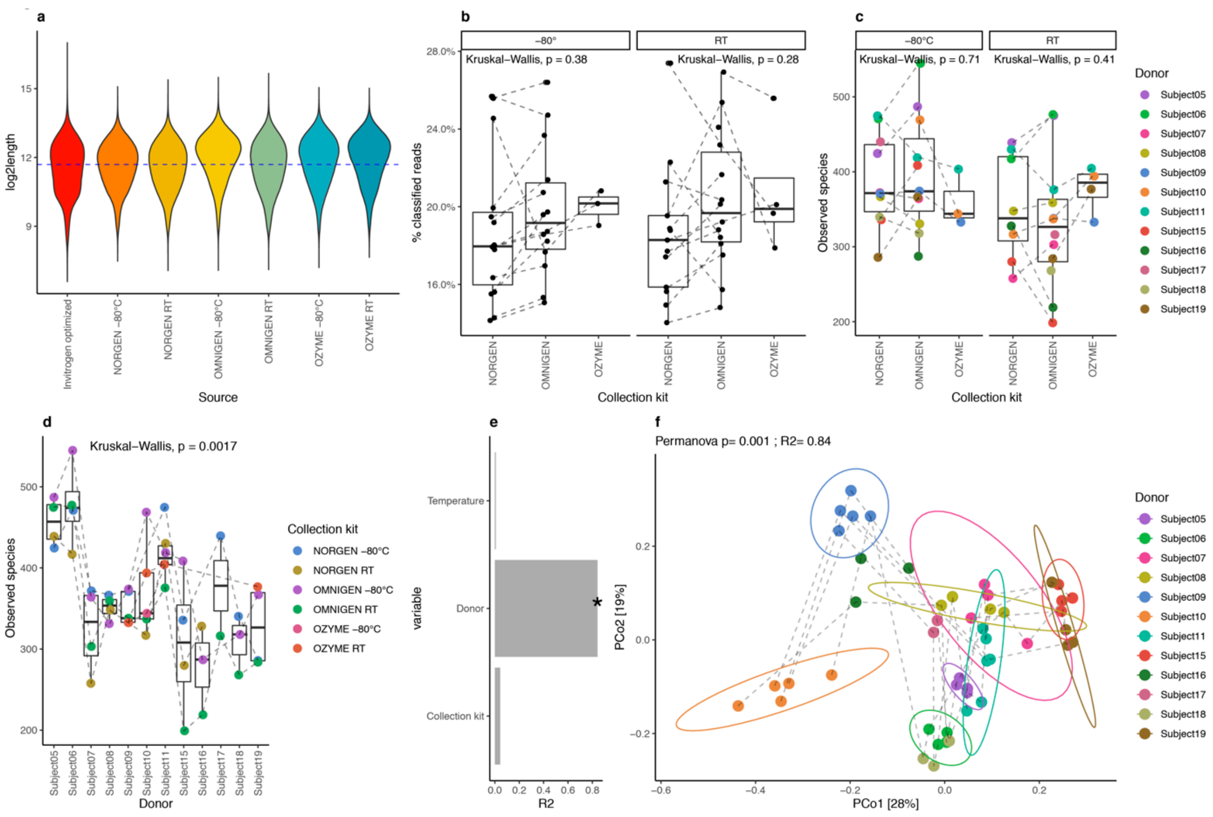

Supplemental Figure S6: Impact of collection kits and storage conditions on metagenomic human stool composition from Nanopore sequencing data. (a) log2-read length distribution of ONT reads across collection kits and temperature storage conditions. For comparison, the log2-read length distribution of initial Invitrogen optimized reads is included. Dashed blue line represents the median log2-read length from the entire dataset. (b) Difference in the fraction of classified reads by Centrifuge strategy between collection kits stratified by storage condition. (c) Differences in microbial diversity between collection kits stratified by storage condition. (c) Differences in microbial diversity (Observed species) between donors of fecal samples in this experiment. (e) Impact of difference experimental variables (donor, temperature, collection kit) over microbiome composition of studied samples. The barplot represents the effect sizes (R2) from Permanova tests of variables in Y-axis over a beta-diversity distance matrix computed from Centrifuge-based genus abundance data (*=P-value<0.05, Permanova test). (F) PCoA ordination of samples from collection kits experiments colored by donor. Dashed lines connect samples collected with same collection kits (Omnigen, Ozyme, Norgen).

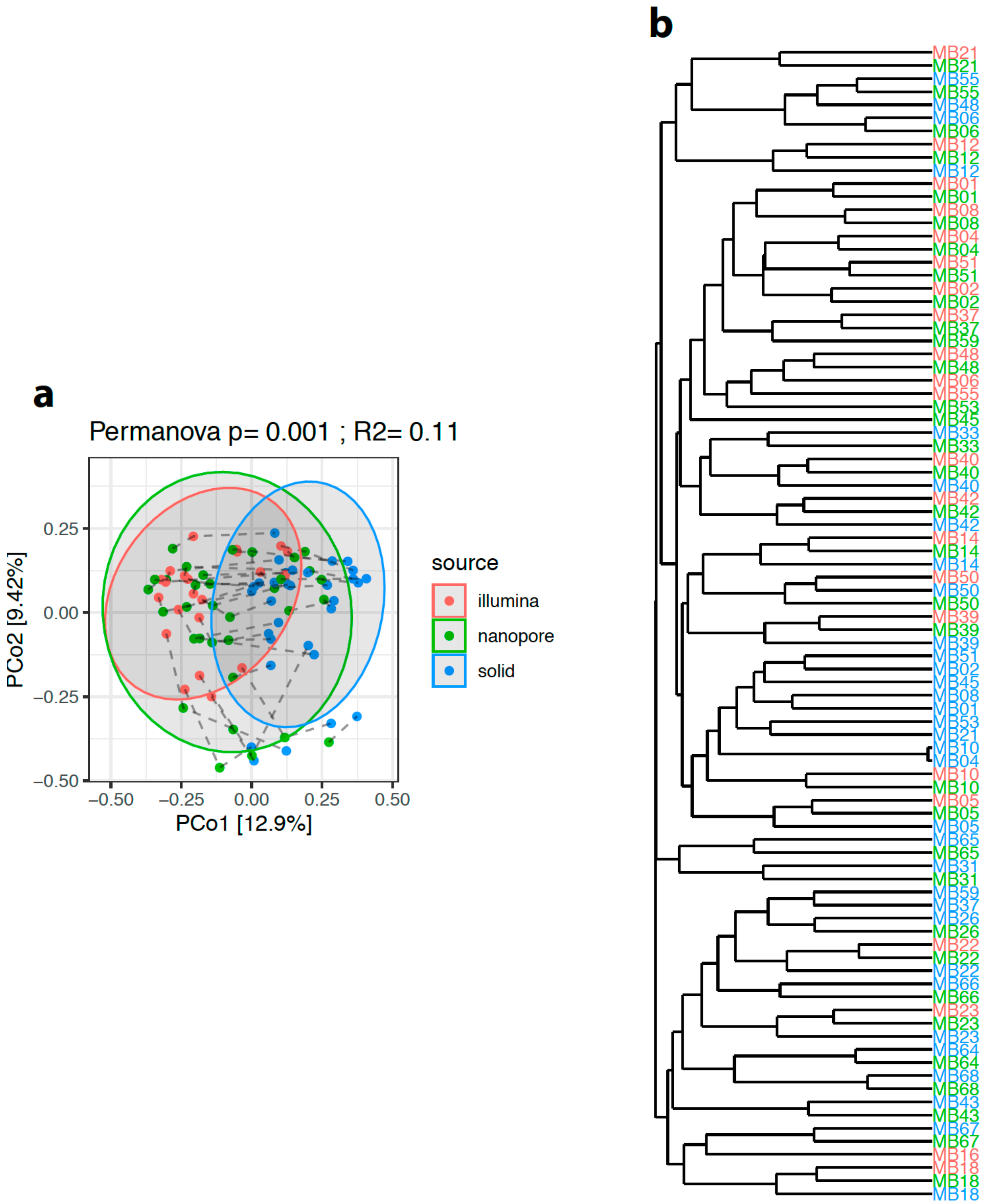

Supplemental Figure S7: Comparison of microbial composition of Microbaria samples between Nanopore, Illumina and SOLiD sequencing data. (a) PCoA of samples from Microbaria study based on genus-level MGS abundance data from three different sequencing technologies (n=33 for Nanopore (ONT) and SOLiD; n=21 for Illumina). Significant effect of sequencing technology in microbiome composition is observed in PERMANOVA test (P-value=0.001; R2=0.11), with sample points from Illumina and ONT sequencing data (both generated with Invitrogen optimized protocol) closer than sample points from SOLiD sequencing (different DNA extraction method) (b) Hierarchical clustering of Microbaria samples product of different sequencing methods based on

same genus-level MGS abundance data as PCoA in panel A. Sample points from Illumina and ONT sequencing over same biological sample tends to cluster together in the dendrogram.

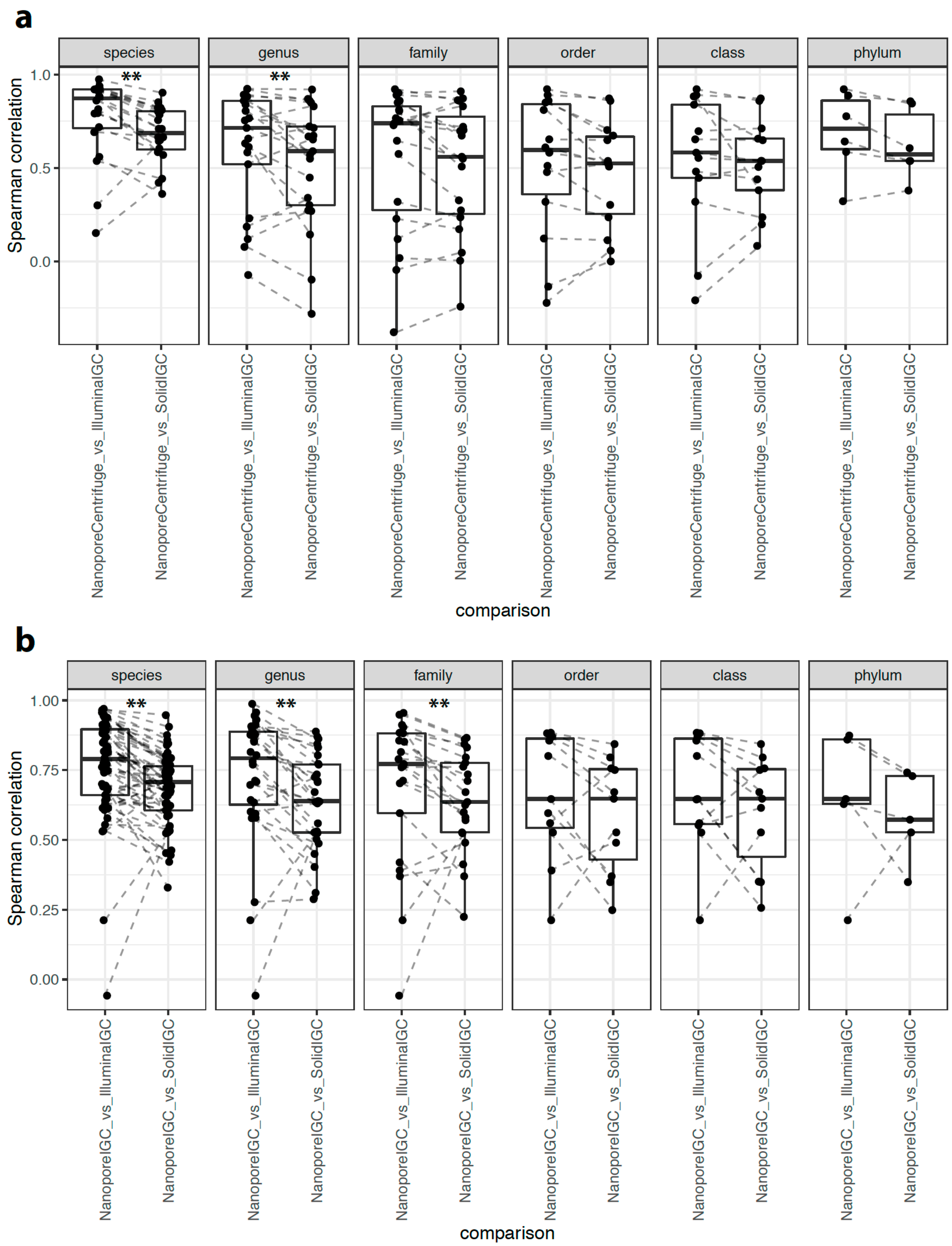

Supplemental Figure S8: Comparison of similarities in the abundance of taxonomic features between Nanopore and SOLiD-Illumina sequencing data. (a) Correlations of taxonomic feature abundances at different levels of taxonomic hierarchy between Nanopore (ONT) abundance data based on Centrifuge approach and Illumina and SOLiD abundance data (based on MGS from IGC catalog). (b) Correlations of taxonomic feature

abundances at different levels of taxonomic hierarchy between ONT abundance data and Illumina and SOLiD abundance data based on MGS from IGC catalog. Dashed lines connect the same taxonomic feature across comparisons. ** P-value<0.01, Paired Wilcoxon rank-sum test.

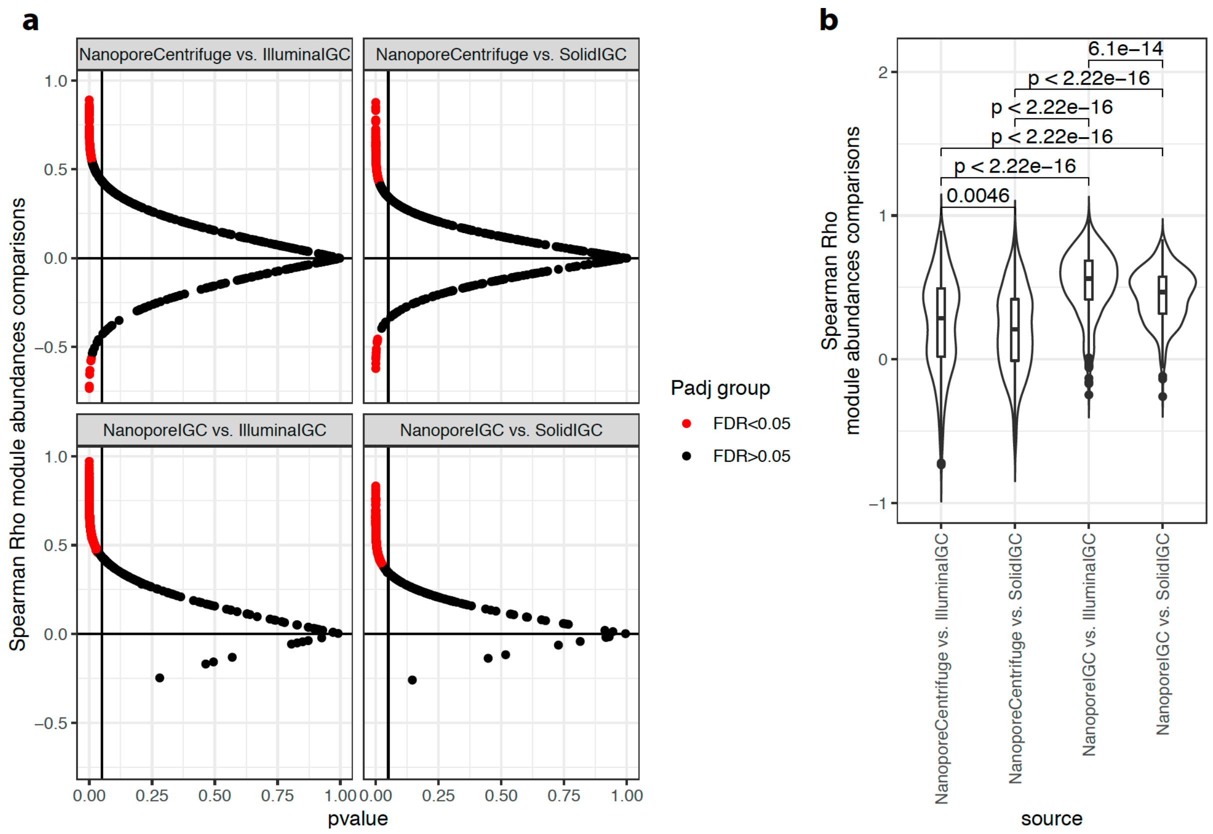

Supplemental Figure S9: Comparison of similarities in KEGG functional modules abundance between Nanopore (ONT) and SOLiD-Illumina sequencing data. (a) Vulcano plots comparing the results of Spearman correlations of individual KEGG functional modules between ONT and Illumina-SOLiD sequencing data. (b) Comparison of similarities in module abundance data (Spearman's Rho) between ONT abundance data (from Centrifuge and from MGS abundance data) and Illumina and SOLiD abundance data (based on MGS abundance data). P-values from pairwise Wilcoxon rank-sum tests of Spearman's rho distributions between comparisons in x-axis are shown above the violin plots.

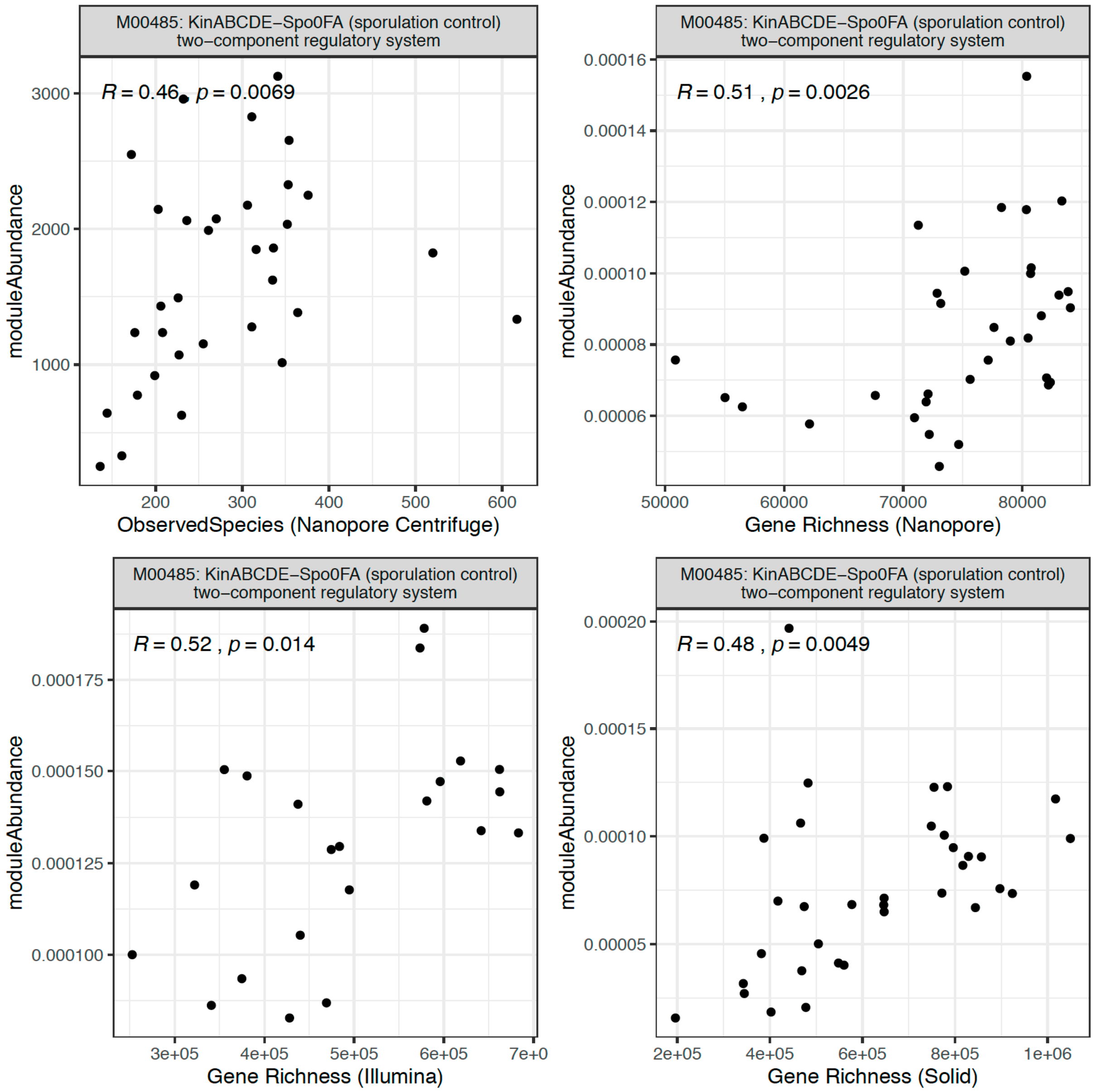

Supplemental Figure S10: Scatterplots of KEGG Sporulation module M00485 abundance and microbial diversity across different quantifications of diversity and module abundance based on Nanopore (ONT), SOLiD and Illumina sequencing data. Results of Spearman correlation tests are shown for each comparison.

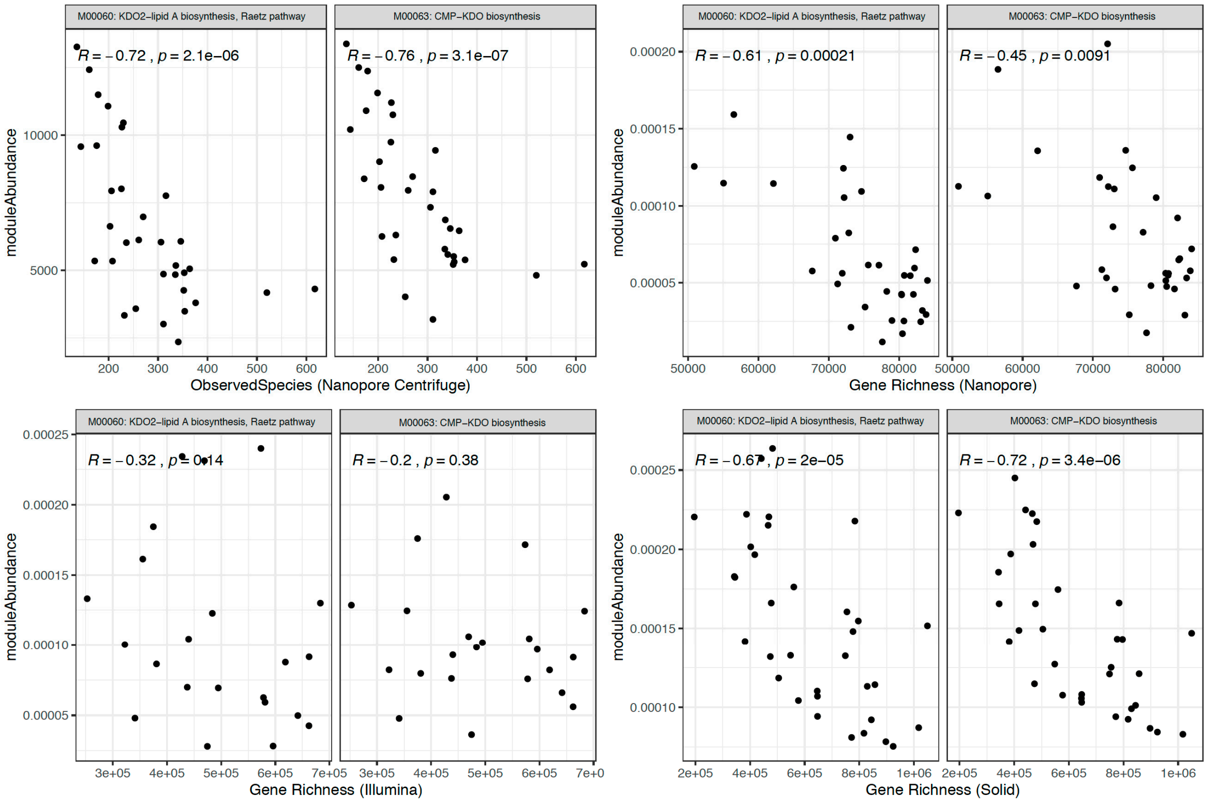

Supplemental Figure S11: Scatterplots of abundances of KEGG LPS biosynthesis modules (M00060, M00063) and microbial diversity across different quantifications of diversity and module abundance based on Nanopore (ONT), SOLiD and Illumina sequencing data**.** Results of Spearman correlation tests are shown for each comparison.
